# Declined Total Fertility Rate Among Immigrants and the Role of Newly Arrived Women in Norway

**DOI:** 10.1007/s10680-019-09541-0

**Published:** 2019-11-06

**Authors:** Marianne Tønnessen

**Affiliations:** 1 Research Department, Statistics Norway, PO Box 8131 Dep, 0033 Oslo, Norway; 2 Department of Sociology, Stockholm University, SUDA, 106 91 Stockholm, Sweden

**Keywords:** Immigrant fertility, Migrant fertility, Migration, Decomposition, Immigration, Fertility

## Abstract

In many Western countries, the total fertility rate (TFR) of immigrant women has declined over the last decades. This paper proposes two methods for investigating such changes in the aggregate immigrant fertility level: what-if scenarios and a formal decomposition. Both methods disentangle the effect of changed composition—by origin area and duration of stay—from the effect of changed fertility within subgroups. The methods are applied to data from Norway, where immigrant TFR declined from 2.6 births per women in 2000 to below 2.0 in 2017. The results show that this decline is not due to successful integration, nor changed composition of immigrant women by origin area or duration of stay. A main reason for the decline is found among newly arrived immigrant women, particularly from Asia. They have a considerably lower fertility now than what the newly arrived had 15–20 years ago. After investigating several possible reasons for the TFR decline among the newly arrived, decreased fertility in origin areas is suggested as a key driver.

**Electronic supplementary material:**

The online version of this article (10.1007/s10680-019-09541-0) contains supplementary material, which is available to authorized users.

## Introduction

**Fig. 1:**
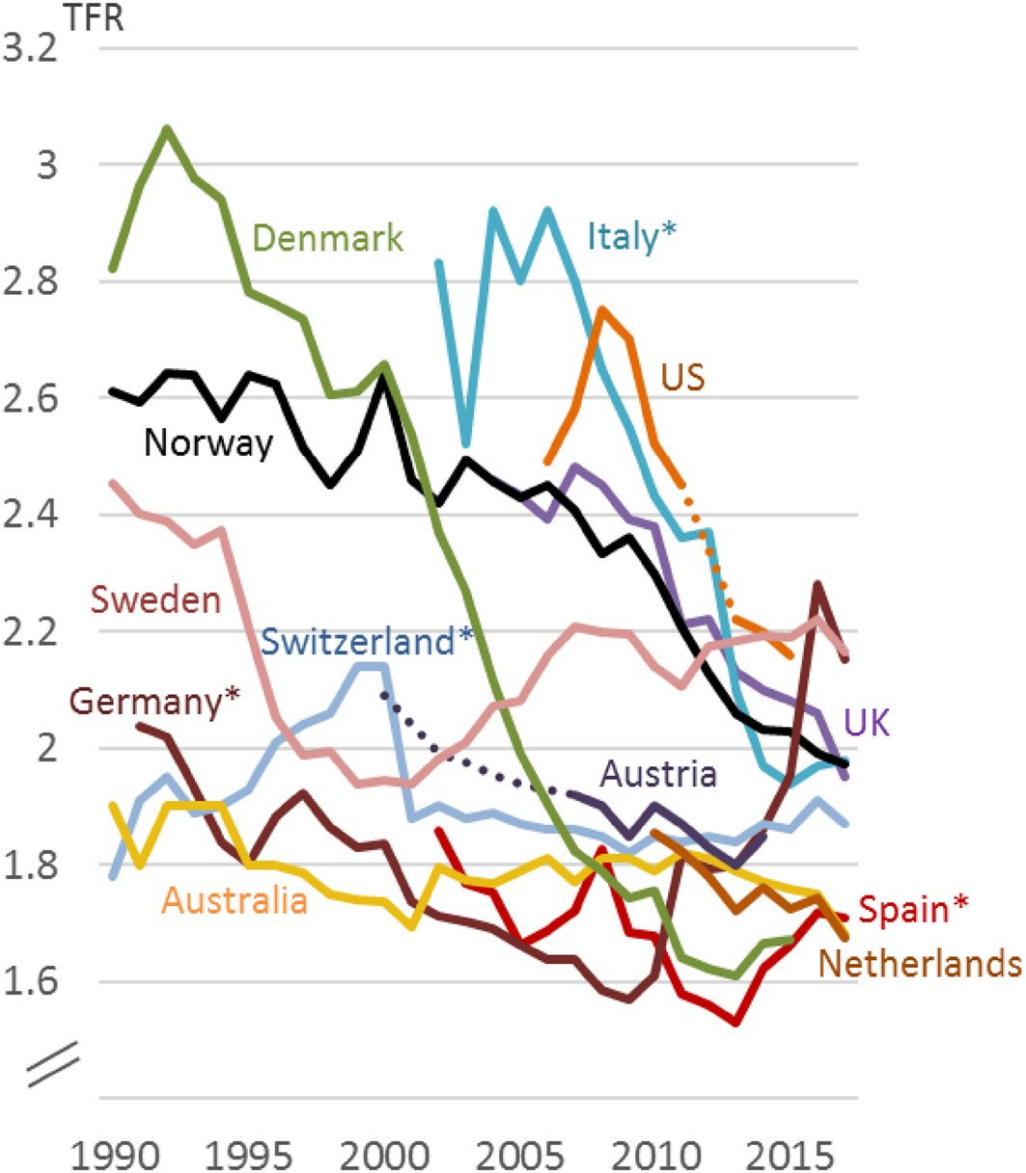
Total fertility rates among immigrant women (or *noncitizen women)^a^ in some Western countries, 1990–2017^b^. ^a^Figures for noncitizens are used when figures for immigrants were not available. ^b^Dotted lines indicate lack of data for some years. *Sources*: Australian Bureau of Statistics (abs.gov.au), Statistics Denmark (dst.dk), Geburtenbaromenter Austria (oeaw.ac.at), Germany’s Federal Statistical Office (destatis.de), Italian National Institute of Statistics (Istat.it), Statistics Netherlands (cbs.nl), Spain’s National Statistics Institute (ine.es), Switzerland’s Federal Statistical Office (www.bfs.admin.ch), UK’s Office for National Statistics (ons.gov.uk), American Community Survey/Center for immigration studies (cis.org), Statistics Sweden and Statistics Norway

The total fertility rate (TFR) of immigrant women has declined in many Western countries, as shown in Fig. 1. The TFR levels vary, which may partly be due to different compositions of immigrant women, but a falling trend is found in most countries. This paper shows how such a decrease in the TFR of immigrant women can be investigated.

Immigrants’ fertility has received attention in many Western countries, for several reasons. Many immigrant women are in their childbearing ages; hence, their fertility has large impact on the number of births. In Western Europe in 2017, where 13% of the population was foreign-born, every fourth birth was to a foreign-born mother (Eurostat 2019a, b). Hence, immigrant fertility affects Western countries’ population size and age composition, which translates into needs for kindergartens and schools, and in the longer run it affects labour force, future number of women in childbearing ages and the old-age-dependency ratio. Immigrant TFR is also relevant for the public debate in many Western countries, where some are concerned about the future number and share of immigrants and immigrants’ children.

Numerous studies have contributed to explaining immigrant’s fertility behaviour, displaying how fertility patterns vary between different groups of immigrant women, for instance by origin area or duration of stay. However, not much attention has been devoted to understanding changes in the aggregate fertility level of immigrants in a country.

The total fertility rate is the most common aggregate measure of fertility, summarizing current fertility patterns into a single number. Figures on immigrant TFR are regularly published in many countries and used by policy makers and others in the public debate. However, there is a risk of drawing too quick conclusions based on this summary measure. For instance, a decreased immigrant TFR may be interpreted as a sign of successful integration of immigrants. However, even if integration often implies that an immigrant woman’s fertility decreases by her duration of stay, this will only lead to decreased TFR for all immigrant women if the *proportion* of women with long duration of stay increases. Another factor that could change immigrant’s TFR is changed composition of immigrant women by origin area, for instance if the proportion of immigrants from low-fertility countries increases. Yet another possible reason could be changed fertility *within* subgroups of immigrant women (by duration of stay and country of origin).

Understanding the determinants of a changed immigrant TFR is essential in order to implement appropriate policy responses and better forecast future fertility. Therefore, the aim of this paper is to show how changes in the overall immigrant TFR can be scrutinized.

The paper is organized as follows: The first part briefly reviews how previous research on migrant fertility has identified two factors as particularly important for immigrant women’s fertility: area of origin and duration of stay in the destination country.

Secondly, two approaches are introduced for disentangling the TFR effect of changed composition by origin area and duration of stay from the effect of changed fertility behaviour within subgroups of immigrant women (by origin area and duration of stay): *what*-*if*-*scenarios* and a *formal decomposition*. These methods have not, to my knowledge, previously been used to investigate immigrant fertility trends. The methods are applied to data from Norway, where TFR among immigrant women decreased from 2.6 births per woman in 2000 to less than 2.0 in 2017.

The two approaches broadly give the same conclusion: Although immigrants’ fertility often declines with their duration of stay, this does not explain why the immigrant TFR in Norway has fallen since 2000, nor does changed composition by origin area. The decrease in immigrant TFR in this period is mainly due to changed fertility *within* subgroups (by origin area and duration of stay). Almost half the decrease is due to the newly arrived immigrant women having a noticeable lower fertility now than the newly arrived had in 2000.

Furthermore, this TFR decrease among the newly arrived immigrant women is decomposed by reason for immigration. The results show that a large part of their decline is linked to the family migrants—women who migrate for family-(re)unification. Their share among all newly arrived immigrants has decreased since 2000, and so has their fertility. Among the newly arrived family immigrants from Asia, TFR declined by more than two births per women.

This TFR decrease among newly arrived family migrants, particularly from Asia, is investigated by exploring other factors such as age at arrival, education, births before migration and whether the male partner was a migrant. The TFR trends of newly arrived family immigrants are also compared with TFR trends in their countries of origin. This latter approach suggests that the fertility decline among newly arrived family migrants from Non-Western countries may, at least partly, reflect a declined fertility in the country of origin. Thus, if fertility continues to decline in high-fertility countries, as the United Nations assumes, the results of this paper suggest that further fertility declines may be expected among newly arrived immigrants from these countries in Western societies.

## Theory and Previous Research

Although the TFR of immigrants is widely produced and used, previous research has not focused much on explaining changes over time in this macro-measure. However, on a more micro-level, substantial work has been done to uncover factors that can explain fertility patterns among different groups of immigrant women in Western countries.^1^

### Hypotheses on Individual Immigrant Women’s Fertility

From this research, two factors appear particularly crucial for an immigrant woman’s fertility: her origin area and her duration of stay. These two factors will play a key role in the methods presented later in this paper. Several hypotheses may explain their importance. A thorough overview of migrant fertility hypotheses are presented in, for instance, Kulu (2005), Kulu and Milewski (2008), Milewski (2010), Kulu and González-Ferrer (2014), Wilson (2015) and Adserà et al. (2015). The hypotheses can broadly be divided into two groups: first, three different hypotheses aim at explaining why immigrants’ fertility tends to change with their *duration of stay*. The hypothesis of interrelated events (or family formation hypothesis) emphasizes that many immigrant women migrate because they are starting a family, so fertility will be particularly high right after migration. The adaptation hypothesis points out that a person’s fertility behaviour is affected by her current context, so when an immigrant settles in a new country, she will adapt over time to this country’s fertility norms. The disruption hypothesis, on the other hand, argues that migration may be stressful and often involves separations of spouses and depressed income, so we can expect a temporary drop in fertility around the time of migration.

All these three hypotheses imply that subgroups with different durations of stay have different fertility norms. Hence, a changing composition of immigrant women by duration of stay would also change the general immigrant TFR.

The second group of hypotheses are concerned with the role of immigrants’ *origin area*. According to the socialization hypothesis, people are formed by their childhood experiences, so that even if they move to a new country, their fertility is defined by the norms and behaviours they once were socialized into. As a complement to this, the selection hypothesis states that immigrants may be a select group compared to nonmigrants in their origin area. Immigrant women’s reason for migration may reveal some of this selection. For instance, women who migrate for work may have lower fertility preferences than an average woman from the same origin area.

When subgroups of immigrant women from different origin areas have different fertility norms, a changing composition of immigrant women by origin area would also change the overall immigrant TFR. Also, if subgroups of immigrant women with different reasons for migration have different fertility norms, a changing composition of immigrant women by reason for migration can affect the TFR for all immigrants.

The significance of duration of stay and origin area is dominant in the literature on migrant fertility. In addition, migrant fertility research has also identified other factors that may affect an immigrant woman’s fertility, such as age at migration, education, residential segregation, whether the male partner is also a migrant and her number of births before migration. Factors like these may explain why we sometimes see changed fertility within subgroups of immigrant women by origin and duration of stay.

### From Micro to Macro

Uncovering factors that affect individual immigrant women’s fertility, which has been the focus in much of the literature in this field, can certainly be used in order to understand macro-trends in immigrant TFR. However, it is not sufficient unless changing composition of immigrant women is also taken into account. For instance, if micro-studies show that immigrant women’s fertility tends to decline by their duration of stay, this does not necessarily translate into a declining TFR for all immigrant women over time unless the *share* of immigrant women with long duration of stay increases. The aim of this paper is to use the factors presented above to identify subgroups of immigrant women whose fertility can be expected to be similar, in order to study how much of the declining macro-TFR for all immigrant women is due to changed composition. Thus, this paper does not test the hypotheses on migrant fertility presented above. Rather, it contributes to the literature by showing how knowledge from micro-studies can be used to investigate changes at the macro-level.

## Data, Measures and Methods

To disentangle changes in the general immigrant TFR, this paper proposes two approaches: *What*-*if scenarios* and a *formal decomposition*. First, in the what-if scenarios, the composition of immigrant women (by eight origin areas and four durations of stay) is allowed to change like it actually did, while the fertility in each subgroup is kept constant at 2000 levels—and vice versa. Second, a decomposition based on Kitagawa (1955) is applied to changing fertility over time.

Both methods address this paper’s main question: To what degree is the decline in immigrant TFR due to changed composition of immigrant women (i.e. by origin area and duration of stay), and to what extent is it due to changed fertility within subgroups of immigrant women? The methods also identify subgroups that are driving the change.

The two methods are demonstrated using register data from Norway. Norway may be a good case for several reasons: As Fig. 1 shows, Norway’s downward trend in immigrant fertility is comparable to many other Western countries’. Also regarding fertility and immigration in general, Norway is similar to many other European countries: The Norwegian TFR in 2000–2017 was higher than the European average, but lower than in countries like France, Iceland and Ireland (Eurostat 2019c). Even if Norway is not a member of the European Union, it is part of Europe’s Schengen Area, where internal border checks have largely been abolished. The share of foreign-born in the Norwegian population has increased markedly in the last decades, and by 2017 it was at 15%. This is higher than the average of 13% in Western Europe, but still lower than in, for instance, Switzerland, Austria, Sweden, Ireland and Belgium (Eurostat 2019a). So with regard to immigration, fertility and immigrant fertility, Norway is comparable to many other Western countries. Another advantage of using Norway as case is the rich Norwegian register data which makes it possible to study how several background characteristics affect immigrants’ fertility.

### Data

The data are from Norway’s population register, which includes complete cohorts of all immigrant women and all their live births in Norway. Immigrants are defined as people born abroad to foreign-born parents and grandparents and who have immigrated to Norway in order to stay for at least 6 months, with legal permission to stay. This study included 207,078 births to immigrant mothers (2000–2017) and a total of 2,773,274 person-years of immigrant women aged 15–49 (< 90,000 yearly in the first years and more than 250,000 in 2017). Due to insufficient information about the mothers, 179 births were excluded from the sample.

### Composition by Origin Area and Duration of Stay

As shown in Fig. 1, TFR among immigrant women in Norway decreased from 2.6 in 2000 to below 2.0 in 2017. The difference between immigrant and native TFR declined as well, from 0.9 to 0.3. Immigrants are also having their children later in life; the fertility decline among immigrant women has primarily been in the younger age groups, so from 2000 to 2017, the immigrants’ age profile of fertility has become more similar to the natives’.

**Fig. 2:**
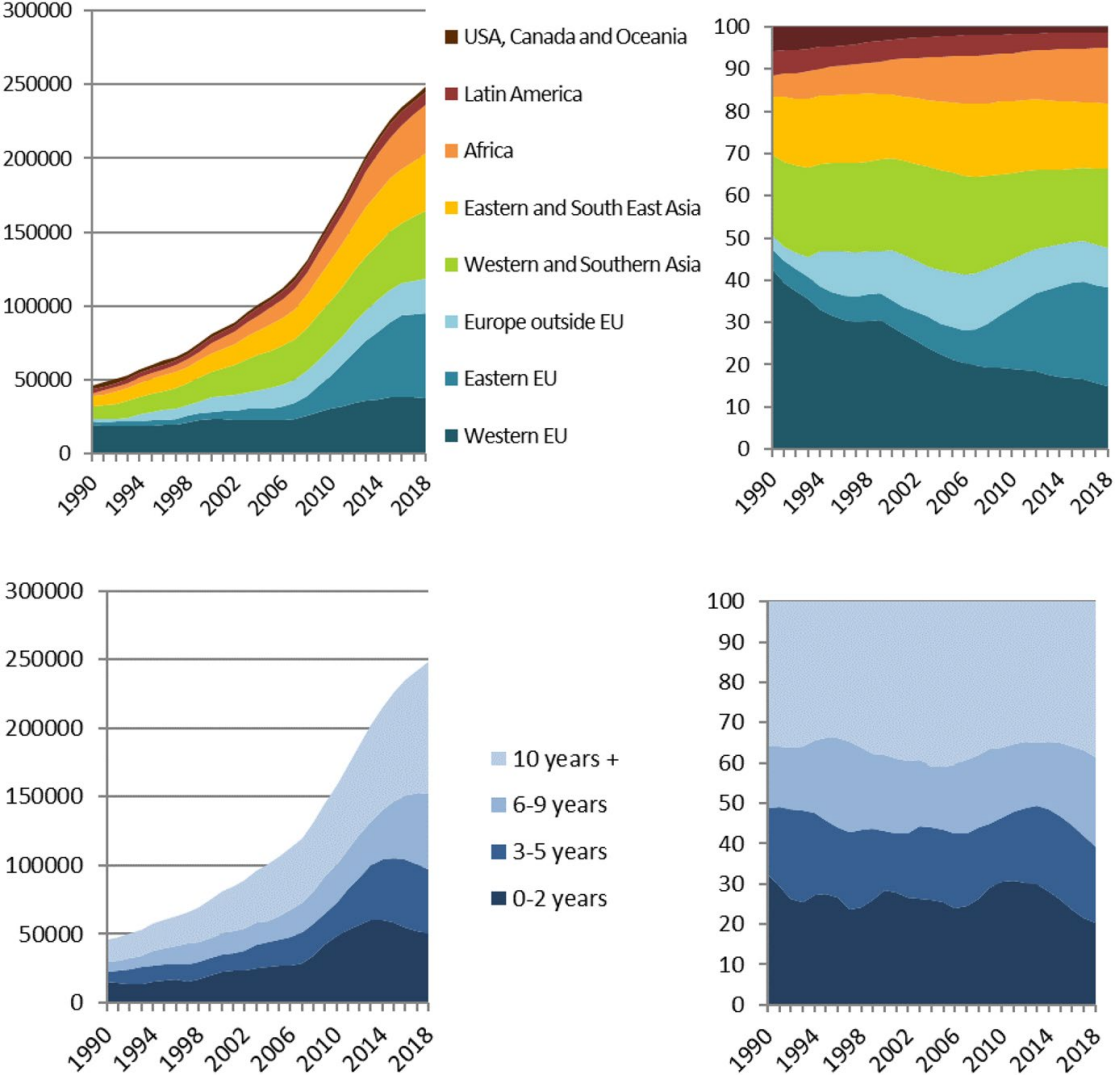
Immigrant women (age 15–49) in Norway, by origin area (upper panel) and duration of stay (lower panel). Absolute numbers (left) and per cent (right). 1990–2018. *Source*: Statistics Norway/own calculations

In this period, both the number and the composition of immigrant women changed markedly. After the European Union enlargement in 2004, a substantial number of women from the new eastern member states migrated to Norway. Immigration from other parts of the world also increased. Figure 2 shows how the numbers and proportions of immigrant women (age 15–49) in Norway changed from 2000 to 2018, by origin area^2^ and duration of stay.^3^

The left panels in Fig. 2 show the absolute number of immigrant women (age 15–49) living in Norway, by origin area (upper panel) and duration of stay (lower panel). The right panels show how the shares in the different groups have changed over time. The number and share of immigrants from Eastern EU has increased substantially, while the share from Western EU has decreased over the whole period. Taken together, the proportion of women from Europe, where fertility is relatively low, only declined marginally from 2000 to 2018.

All the four duration-of-stay groups have seen large increases, whereas the proportions in each group have been relatively stable over the last decades (lower right panel). Thus, the share of women from traditionally high versus low-fertility areas of the world has not changed very much, nor has the distribution by duration of stay. This suggests that changed composition by origin area or duration of stay may not be the main driver behind the immigrant TFR decline.

### The Total Fertility Rate

The total fertility rate (TFR) is the core measure in these analyses. TFR is probably the most widely used fertility measure worldwide (Bongaarts et al. 1998). It is the sum of age-specific fertility rates (ASFR), which are calculated by dividing the number of children born in a certain year to women in a certain age group by all women in that age group.

Although TFR is widely used, it also has some problematic sides. Tempo effects in fertility, such as postponement of births, may have large impact on the TFR even if completed fertility proves to be unchanged (Ryder 1956). Also, as a measure that covers all women in a certain group, the TFR can mask large fertility differences within the group.

TFR is often interpreted as “number of children per woman”. This interpretation is not necessarily fruitful when TFR is used to analyse immigrant fertility. As several authors have noted (for instance, Wilson 2015; Robards and Berrington 2016), TFR may not be a good predictor of completed family size of immigrants, because of distortions in childbearing around the migration event. Hence, in this study, TFR can best be viewed as a measure of *birth intensity* in a certain subgroup in a certain year, and not as an indication of future family size (since, for example, no immigrant woman will have a duration of stay of 0–2 years all her life).

Using TFR as a measure of birth intensity in a certain group at a certain time—rather than as some indication of expected family size for this group—is not so common. If the aim of this study had been to analyse whether immigrants converge to natives’ completed fertility, other measures might have been more appropriate, such as children ever born. All measures of fertility have strengths and weaknesses, which is particularly true when analysing immigrants’ fertility adaptation, because convergence to the native level in one indicator (e.g. TFR) does not necessarily imply convergence in another (e.g. completed family size/cohort fertility) (Tønnessen et al. 2019). However, since the aim of this paper is to investigate changes over time in immigrants’ overall TFR, the TFR and ASFRs are also the measures used in the methods presented.

Tempo effects are shown to be significant for immigrant fertility, and they are often related to the migration event (Andersson 2004). By using separate TFRs for subgroups of women with different durations of stay, the methods in this paper address this kind of tempo challenges associated with the TFR. Also, by using different TFRs for women of different origins, some of the fertility differences among immigrant women—which are masked by the general immigrant TFR—are accounted for.

**Fig. 3:**
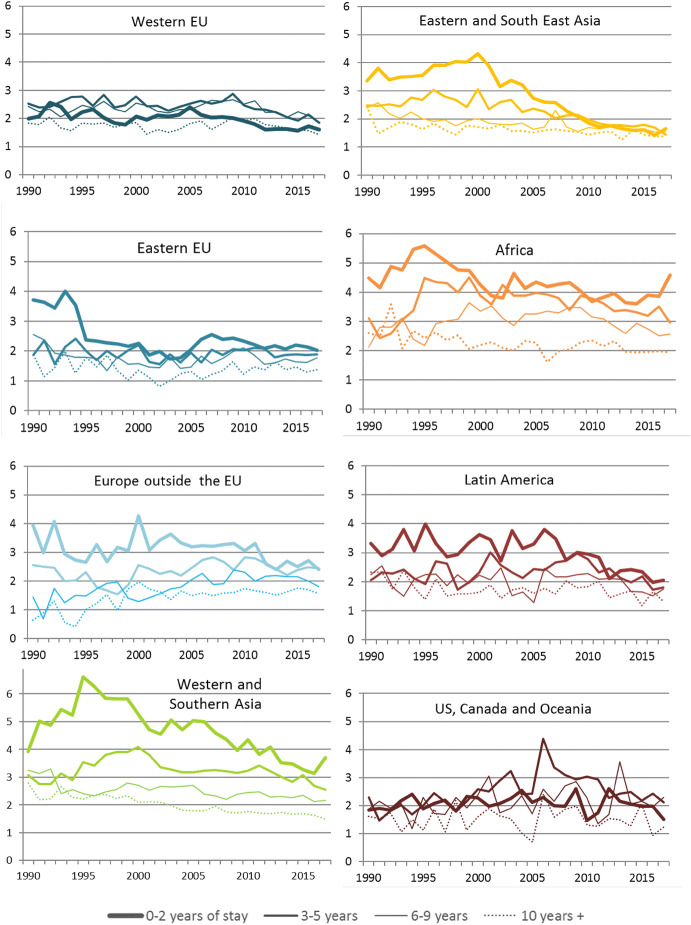
Total fertility rates among immigrant women in Norway, by origin area and duration of stay, 1990–2017 *Source*: Statistics Norway/own calculations

To show how the birth intensities differ between subgroups of immigrant women by origin area and duration of stay, annual TFRs are calculated for all immigrant women aged 15–49 in Norway by eight areas of origin and four durations of stay—altogether 32 subgroups. In the calculations of the underlying ASFRs, five-year age groups were used, since some subgroups of immigrant women are small. The results are shown in Fig. 3. For each origin area, women are grouped by their duration of stay, and they will transfer from one group to another (towards thinner lines) the longer they stay in Norway.

Figure 3 shows three main features. First, TFR is often higher among immigrants from high-fertility areas of the world, such as Asia, Africa and Latin America, in line with the socialization hypothesis. Second, TFR is often highest among women with short duration of stay. This is in line with hypotheses on interrelated events and/or adaptation. Third, some of the lines have quite strong trends, showing a declined fertility *within* subgroups, particularly for newly arrived women from many Non-Western parts of the world.

### Method 1: What-If Scenarios

What-if scenarios can illustrate how these three main features have affected the general immigrant TFR. In these scenarios, certain factors are kept constant while others are allowed to change over time. First, the composition of immigrant women (by origin area and duration of stay) is allowed to change like it actually did from 2000 to 2017 while fertility within each of the 32 subgroups is kept constant at the 2000 level. Second, the composition of immigrant women is kept constant while fertility within each subgroup is allowed to change. This method takes advantage of the fact that TFR across several groups of women can be calculated in this way:

$${\text{TFR}}_{t} = \mathop \sum \limits_{a} {\text{ASFR}}_{at} = \mathop \sum \limits_{a} \frac{{B_{at} }}{{W_{at} }} = \mathop \sum \limits_{a} \frac{{\mathop \sum \nolimits_{i} \left( {{\text{ASFR}}_{ait} \cdot W_{ait} } \right)}}{{W_{at} }} = \mathop \sum \limits_{a} \mathop \sum \limits_{i} \left( {{\text{ASFR}}_{ait} \cdot w_{ait} } \right)$$

where *t* is year, *a* is age, *i* is immigrant group, *B* is the number of births, *W* is the number of women and *w* is the share of all immigrant women (in that age group) who are in group *i*.

Using the last term of this equation, it is possible to keep ASFR_*ait*_ constant at the 2000 level while letting *w*_*ait*_ change. This gives the what-if scenario where only composition is allowed to change. Letting ASFR_*ait*_ change while the *w*_*ait*_ is kept constant gives the scenario where only fertility within each group is allowed to change. In this scenario, the number of women in each age group is fixed as well.

It is also possible to allow fertility to change only within certain groups of immigrant women, keeping both composition and other groups’ fertility constant. This is done to investigate the separate effect of changed fertility among newly arrived immigrant women.

### Method 2: Formal Decomposition

What-if scenarios are well suited to answer hypothetical questions. However, the estimated hypothetical changes in the what-if TFR paths do not necessarily add up exactly to the real TFR change in the same period. A decrease in TFR has one rate component (assuming no change in composition) and one composition component (assuming no change in rates), and also an interaction component reflecting changes in both rates and composition (see elaboration in “Appendix 1”). This can be accounted for with many different methods (Canudas Romo 2003). The decomposition method used in this paper builds on Kitagawa (1955) and the elaboration in Preston et al. (2001, p. 28). In short, if a rate $R = A \cdot B$ and we want to decompose a change in *R*, then $\Delta R = \left( {\Delta A \cdot \bar{B}} \right) + \left( {\Delta B \cdot \bar{A}} \right)$, where ∆ denotes change and $\bar{A}$ and $\bar{B}$ are the mean values of *A* and *B*. In this case, the changes are decomposed into

$$\Delta {\text{TFR}} = \mathop \sum \limits_{a} \mathop \sum \limits_{i } \left[ {\left( {\Delta w_{ai} \cdot \frac{{{\text{ASFR}}_{ai2000} + {\text{ASFR}}_{ai2017} }}{2}} \right) + \left( {\Delta {\text{ASFR}}_{ai} \cdot \frac{{w_{ai2000} + w_{ai2017} }}{2}} \right)} \right]$$

where the first part is the change in a subgroup’s share among all women (in that age group), weighted by the average fertility in that subgroup, and the last part is the change in the ASFR for each subgroup, weighted by that subgroup’s average share of all women (in that age group). The first part is the contribution to overall TFR change from changed composition, whereas the last part is the contribution from changed fertility within the subgroups. For each age group and year, *w* sums to one over all *i*.

Further decompositions by new variables can also be done. To investigate possible selection effects, the above framework is used to decompose the changes in TFR among newly arrived immigrant women by their reason for immigration.

For this paper, the analyses were conducted using the SAS software. The programs used for the what-if scenarios and the decomposition in Table 1 are available upon request (or as online supplementary files?). This study is produced under the Norwegian Statistics Act where privacy concerns restrict the availability of the data sets. Unidentifiable data can be available from the author conditional on permission from Statistics Norway.

**Table 1:**
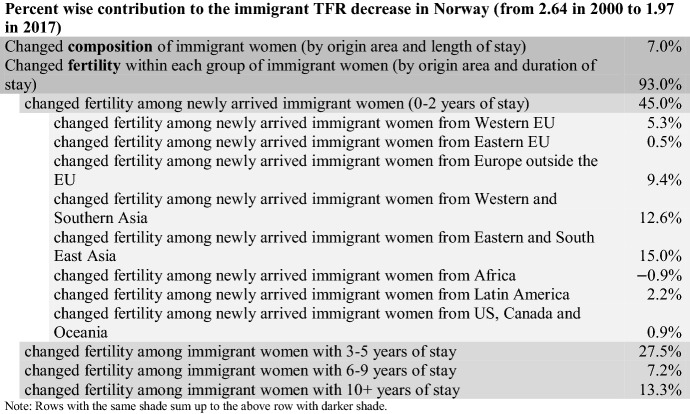
Decomposition of changed TFR among immigrant women in Norway (2000–2017)

## Results

### What-If Results

**Fig. 4:**
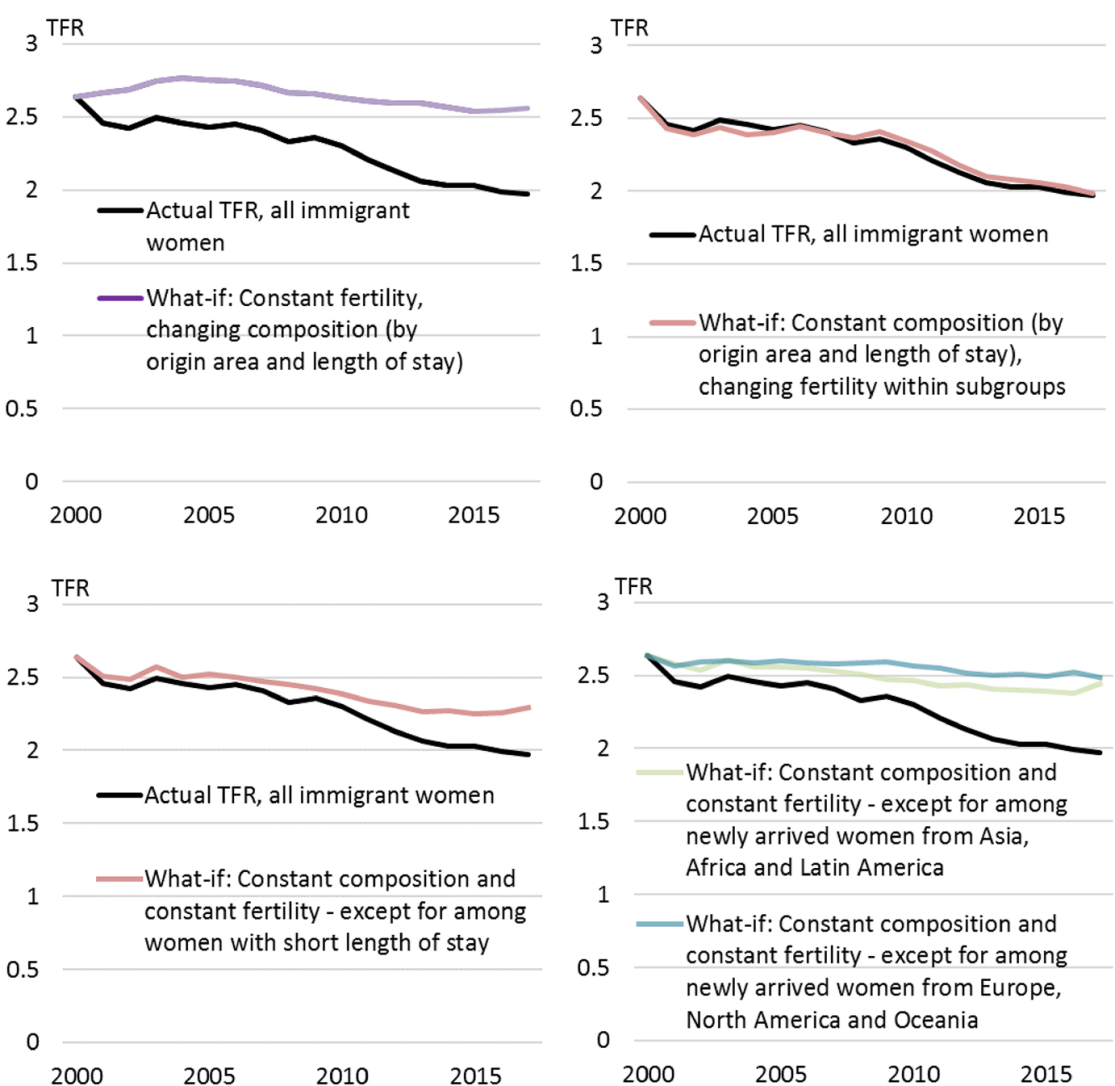
What-if scenarios, where either composition of immigrant women, fertility for all subgroups or fertility for certain subgroups was allowed to change while the other factors were fixed at 2000 level

In the first what-if scenario, fertility in all the 32 subgroups was fixed at the 2000 level, while the composition of immigrant women (by origin area and duration of stay) was allowed to change like it actually did between 2000 and 2017. The resulting what-if TFR for all immigrant women is shown in the upper left panel of Fig. 4. This scenario shows almost no decrease, while the observed immigrant TFR decreased. This is not surprising; as Fig. 2 shows, the shares of women from traditionally high- versus low-fertility origin area, as well as by different durations of stay, were relatively stable from 2000 to 2017. The difference between this what-if scenario and the actual situation can be translated into 600 births in 2001, increasing to almost 4000 births in 2017. Taken together, the fact that the fertility in each subgroup *did* change over this period resulted in almost 35,000 fewer births than in this hypothetical situation with a fertility fixed at the 2000 level.

The upper right panel of Fig. 4 shows the opposite scenario, where composition was fixed at the 2000 level and only fertility within each subgroup was allowed to change. This scenario seems to catch a lot of the changed immigrant TFR, as it closely follows the actual TFR trend registered for immigrants in Norway.

Many of Fig. 3’s panels show a particularly large fertility decrease among women with short duration of stay (0–2 years). To isolate the effect of this decrease, a what-if scenario was calculated where only fertility of newly arrived immigrant women was allowed to change, while all other immigrant women’s fertility, as well as their composition, was kept constant. The results are shown in the lower left panel of Fig. 4. A large part of the total decrease appears to be due to this decline in newly arrived immigrants’ fertility.

The decrease among the newly arrived seems most pronounced among women from high-fertility areas of the world (Fig. 3). The effect of this decrease was explored by creating two what-if scenarios where everything was kept constant except the fertility among newly arrived immigrant women from Asia, Africa and Latin America, and from Europe and USA, Canada and Oceania, respectively. The results are shown in the lower right panel of Fig. 4. Newly arrived immigrants from Asia, Africa and Latin America account for more of the decrease than newly arrived Western immigrants. However, the latter also contribute to the general TFR decline, but not until after 2009.

### Decomposition Results

The decomposition shows that 93% of the TFR decrease among immigrant women in Norway can be attributed to lower fertility *within* the subgroups, while 7% is due to changed composition by origin area and duration of stay (Table 1).

The fertility change among the newly arrived immigrant women accounts for 45% of the TFR decrease for all immigrant women in Norway since 2000. The contribution is particularly large among newly arrived immigrants from Asia, who have a considerably lower fertility now than what the newly arrived from Asia had in 2000 (Fig. 3). The newly arrived Asian women alone account for 27.6% of the TFR decrease for all immigrant women in Norway since 2000.

The fertility decline among women with somewhat longer duration of stay (3–5 years) also plays a role; 27.5% of the total decline can be attributed to this group. About half of this (13.4%) is due to lower fertility among Asian immigrants.

### Further Decomposition of the TFR Decline Among the Newly Arrived

To sum up, both the what-if scenarios and the decomposition suggest that the decline in immigrant TFR in Norway to a large extent is due to the newly arrived immigrant women having a lower fertility now than the newly arrived had in 2000.

Some of this decrease may be due to changed selection. For instance, reasons for migration may have changed. Reason for immigration is recorded at immigrants’ first arrival in Norway (unless they are Nordic citizens). Research from other countries has shown that fertility tends to differ by reason for migration, and women who migrate for family reasons often have relatively high fertility (Castro Martín and Rosero-Bixby 2011; Mussino and Strozza 2012; Ortensi 2015). As further documented in “Appendix 2”, family migrants are found to be essential for understanding the TFR decrease among all immigrants in Norway, in two ways: Their proportion among all newly arrived immigrant women has decreased for many of the origin groups, and their fertility has declined in all origin groups. Among the newly arrived family migrants from Asia, TFR fell by more than two births per woman (from 6.5 to 4.3 among Western and Southern Asians, and from 5.1 to 2.9 for Eastern and South East Asians, see Appendix Fig. 7).

Results from the decomposition by reason for migration are summarized in Appendix Table 2, which is an extension of Table 1 where the contribution from newly arrived immigrant women in each origin group is further broken down. The two groups of newly arrived family immigrants from Asia, who had the largest TFR decrease, also made the largest contribution to the declined TFR for all immigrants. Among newly arrived immigrants from Western and Southern Asia, lower fertility among family migrants accounts for 9.7% of the overall TFR decline. Similarly, lower fertility among newly arrived family migrants from Eastern and South East Asia accounts for 8.2%. Hence, decreased fertility among newly arrived family migrants from Asia alone accounts for 18% of the TFR decrease of all immigrant women in Norway, which is a large effect from a relatively small group—by end-2017, they constituted 3% of all immigrant women in childbearing ages (5% in 2000).

**Table 2:**
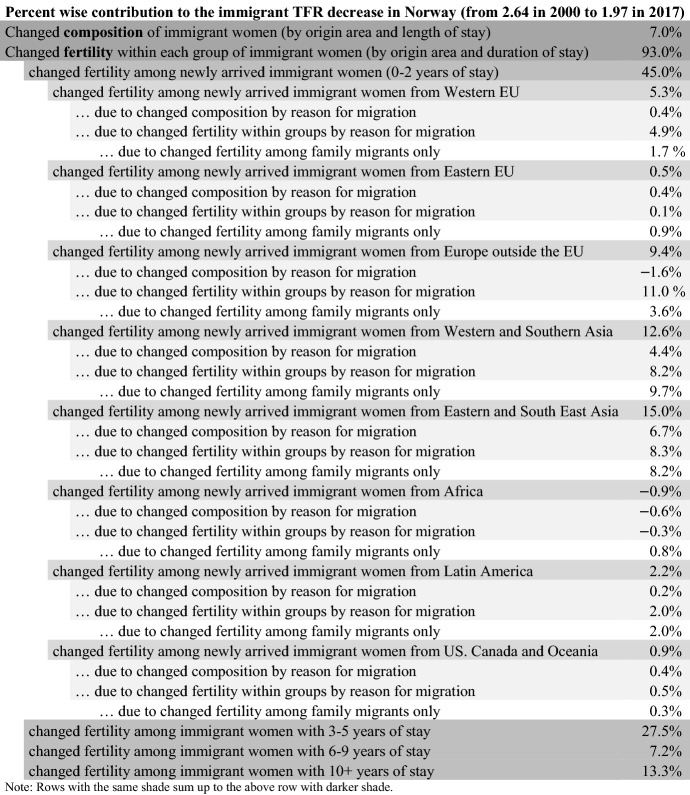
Further decomposition of the TFR change among newly arrived immigrant women in Norway 2000–2017, by reason for migration

### Possible Reasons for the Decline Among the Newly Arrived

As the results show, after taking into account two of the most important determinants of immigrant fertility—duration of stay and area of origin—one group is identified as essential for the TFR decrease among immigrants in Norway: the newly arrived immigrant women. After also taking into account the reason for migration, family migrants, particularly those from Asia, are found to be key drivers.

This section investigates several possible reasons why the TFRs of newly arrived immigrants, and especially family migrants from Asia, have changed. Previous studies have shown that several other factors may influence immigrants’ fertility, such as age at migration, education, residential segregation, whether the male partner is also a migrant and the number of pre-migration births. Changes in the general Norwegian context or in the immigrants’ origin areas may also be important.

First, the trend could be *part of a general fertility decline* in Norway. However, TFR among native women increased in part of this period (2002–2009). Moreover, Fig. 3 shows that immigrants with longer duration of stay do not display a similar trend as the newly arrived.^4^

Second, *age of arrival* is found to be crucial for an immigrant woman’s fertility, indicating that immigrants who arrive as children may adapt faster [shown for instance by Adserà et al. (2012) for fertility patterns in Canada, USA and France]. However, none of the newly arrived immigrant women (0–2 years of stay) have been able to spend much of their youth in Norway.

Third, the fertility decline among newly arrived migrants could be due to *a changed timing of births* after or before the migration. The number of pre-migration births has been shown to affect immigrants’ fertility in other countries (Toulemon 2004; Persson 2013; Choi 2014; del Rey and Grande 2015; Robards and Berrington 2016). Women migrating to Norway may to an increasing extent have given birth before migration and bring their children from abroad instead of giving birth in Norway. However, the number of immigrating children (age 0–15) has evolved similarly to the number of immigrating women (age 15–49) in this period, indicating that each arriving woman does not bring more children to Norway. Alternatively, circumstances around the migration event may have led to more postponement of births. This would imply that fertility among immigrant women with slightly longer duration of stay would increase after some years. However, fertility has also fallen among women with 3–5 years of stay (Table 1).

Forth, several studies have found an effect of *education* on immigrants’ fertility; immigrant women with higher education tend to have lower fertility (Kahn 1994; González-Ferrer et al. 2017). Thus, a higher share of more educated immigrant women would suggest a lower TFR. However, the proportions of high and low educated women evolved quite similarly from 2000 to 2017, whereas the TFR within each of these groups declined markedly, suggesting that educational composition does not explain the TFR decrease.

Fifth, *residential segregation* or *immigrant density* may influence immigrant women’s fertility (Lichter et al. 2012; Wilson and Kuha 2017); if they live in less segregated areas, their fertility is often closer to the natives’. However, most municipalities in Norway had a higher density of foreign-born from most origin areas in 2017 than in 2000 (Statistics Norway 2019). From this, we would actually expect an increased immigrant TFR.

Sixth, *changing Norwegian immigration policies* could explain some of the changes, most notably the lower share of family migrants among the newly arrived. From May 2003, immigrants admitted to Norway following application for political asylum were no longer exempt from subsistence requirements when reuniting with their spouses. Later the family unification requirements were further tightened (Brochmann et al. 2011). Effects of the May 2003 change were assessed by Bratsberg et al. (2010), who found that it curbed family reunions. Thus, policy changes probably contributed to the decreased share of family migrants, but it is an open question whether it had any effect on the family migrants’ fertility.

The family migrants’ fertility may have declined due to *changed background of the child’s father*. Research from Norway and elsewhere shows that people with immigrant background often have higher fertility if their partner is also an immigrant (Mohn 2016; Van Landschoot et al. 2017), and immigrant women who prefer a Norwegian partner may also have fertility preferences closer to the Norwegian level. However, the share of births among all newly arrived immigrant women where the father was Norwegian, decreased rather than increased after 2000.

**Fig. 5:**
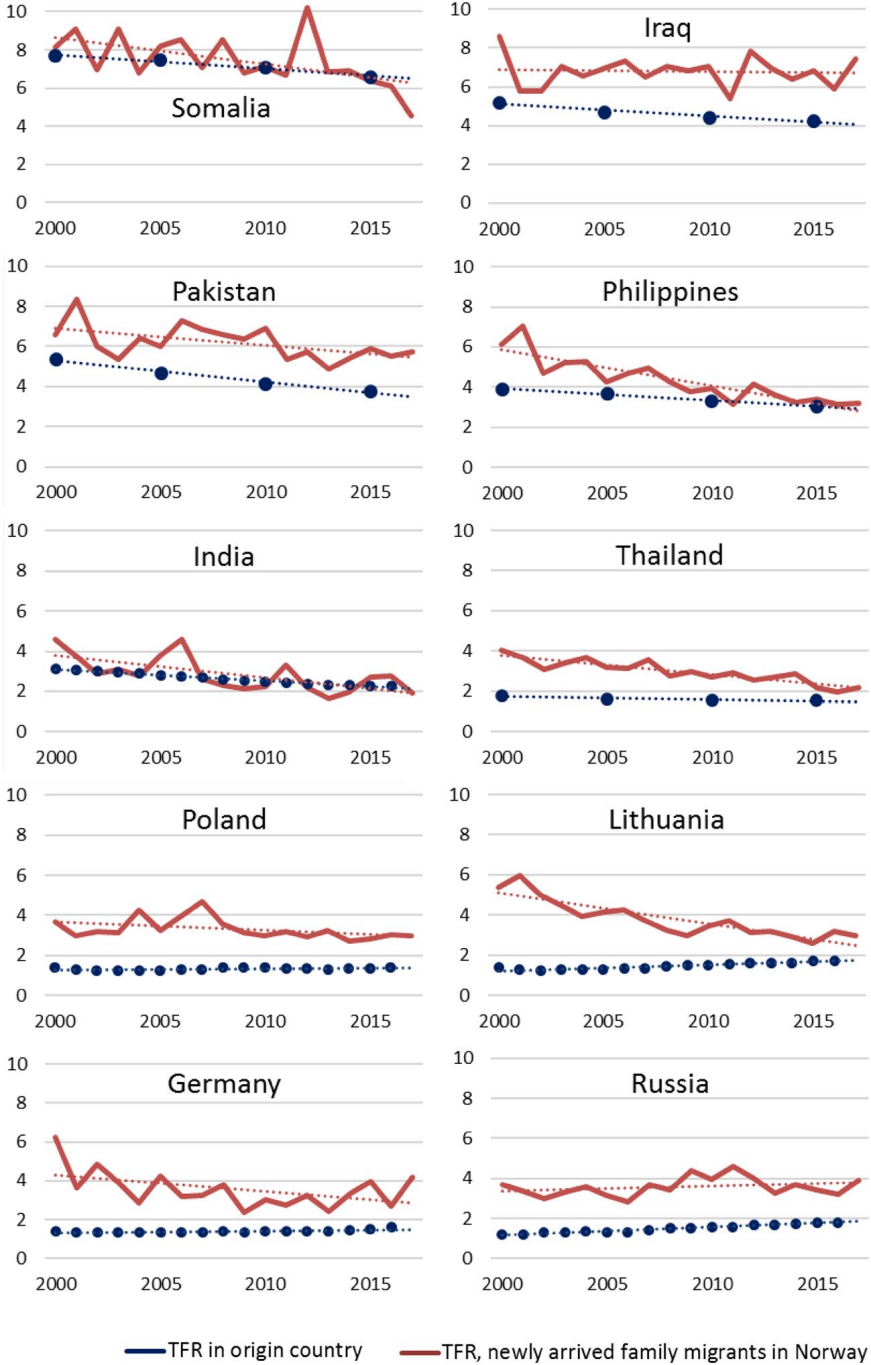
Total fertility rates among the largest groups of newly arrived family immigrant women in Norway and in their origin countries (linear trends in thin dotted lines), 2000–2017. *Sources*: Human Fertility Collection (Russia, India), Eurostat (Poland, Lithuania, Germany), United Nations (Somalia, Iraq, Pakistan, Philippines, Thailand) and Statistics Norway/own calculations. The UN data are given for 5-year intervals; in these graphs, they are plotted at the last year of the interval

Finally, fertility among newly arrived family migrants may have declined because of *declined fertility in origin areas*. In many Non-Western parts of the world, fertility is noticeably lower today than in 2000 (UN 2019). Hence, the newly arrived immigrant women from these areas grew up in societies with different fertility norms than those who arrived one or two decades before, implying that socialization has changed. In Fig. 5, fertility among newly arrived family migrants in Norway from the main origin countries is combined with data showing the TFR in their origin countries. Although the levels differ (which may indicate selection, since these women migrate to start a family), the trends for several of the Non-Western groups show the same direction as the trend in their origin country. This suggests that origin country fertility trends may indeed matter for the fertility of newly arrived immigrant women, at least for family migrants from countries where fertility has been high.

## Discussion

At first glance, declined TFR among immigrant women might easily be interpreted as a sign of successful integration of immigrants. There are, however, reasons to be careful before drawing such conclusions. Although immigrant women with long duration of stay often have lower fertility than the newly arrived, this can only explain an overall TFR decrease if the share of immigrant women with long duration of stay increases.

After investigating possible compositional reasons for the TFR decline among immigrants in Norway, such as changed composition by origin area (which matters according to the socialization hypothesis), changed composition by duration of stay (which matters according to the adaptation, interrelation of events and/or disruption hypotheses) and changed composition by reason for immigration (which matters according to the selection hypothesis), fertility changes are still clearly seen among immigrant women in Norway. In particular, TFR declined markedly among newly arrived family migrants, particularly from Asia (and Latin America). This seems to be related to fertility trends in origin areas. Such origin area trends are sometimes overlooked in studies and theories of immigrant fertility. Although the newly arrived immigrant women grew up in the same origin areas as those who moved to Norway one or two decades ago, they grew up in a different time. And as societies change over time, so does socialization.

This study has relevance for research on migrant fertility as well as for policy. First, it proposes methods to investigate changes in the overall immigrant TFR in a country by disentangling composition effects from the effects of changed fertility within subgroups. The methods can be used in any context with adequate data on births and mother’s characteristics.

Second, the results show that the decreased immigrant TFR in Norway is mainly driven by lower fertility among newly arrived women—possibly partly reflecting declining fertility trends in their countries of origin. This may remind migration researchers to look for explanations of changed immigrant fertility beyond the destination country and characteristics of the individual migrants. Moreover, it points to the need for immigrant fertility studies to take into account time *of* arrival as well as time *since* arrival, particularly when there have been clear trends in origin area fertility. For instance, one should be cautious when pooling immigrant women over many arrival cohorts unless changing fertility in origin is controlled for.

Third, this study can be a reminder for policy makers and others not to draw too quick conclusions about the effect of domestic policies on immigrant TFR. Although an immigrant woman’s fertility often declines with her duration of stay, due to, for instance, successful integration, this does not necessarily translate into a declining TFR for all immigrants.

Fourth, the results of this study also point to the future: if changed fertility in origin areas is a driver behind the fertility decline among many Non-Western newly arrived migrants, and if fertility continues to fall in important origin areas—which the UN projects for high-fertility parts of the world (UN 2019)—we may expect further fertility declines among immigrants from these areas. Moreover, policies affecting fertility preferences in high-fertility parts of the world may, in turn, affect the fertility of Western countries’ own immigrant populations.

## Conclusion

Immigrants’ total fertility rate has declined in many Western countries over the last decades. This may be due to several factors, such as successful integration, changed composition of immigrants by origin area, or other reasons. Whereas existing research has focused mainly on individual immigrant women’s fertility behaviour and variations between groups of immigrant women, there is much less evidence on the mechanisms behind changes in the aggregated fertility level of all immigrants in a country. This paper aims at filling the knowledge gap by proposing two methods—what-if scenarios and a formal decomposition—to disentangle the effect of changed composition from the effect of changed fertility within subgroups.

Both methods are demonstrated using data from Norway, where immigrant TFR declined from 2.6 in 2000 to below 2.0 in 2017. The effect of changed composition by origin area and duration of stay was disentangled from the effect of changed fertility within subgroups (by origin area and duration of stay). The results show that although an immigrant women’s fertility often declines with her duration of stay, this is not the main reason for the TFR decrease, nor is changed composition by origin area. Instead, most of the TFR decline is due to changed fertility within the subgroups, most notably among the newly arrived immigrant women, who have lower fertility now than the newly arrived had 15–20 years ago. In particular, lower fertility among newly arrived immigrant women from Asia accounts for 27.6% of the TFR decline among all immigrant women.

This fertility decline among newly arrived women was further decomposed by reason for migration, and family migrants appear to provide a key: their share among all newly arrived immigrant women declined in this period, and so did their fertility. After investigating several possible reasons for their fertility decline, such as education level, age at migration, number of pre-migration births and residential segregation, I suggest that a large part of the fertility decline among newly arrived family migrants from Non-Western parts of the world may be a reflection of fertility decline in origin areas. Consequently, if fertility continues to decline in high-fertility countries, this may bring about further fertility declines among newly arrived immigrants from these countries in Western societies.

### Electronic supplementary material

Below is the link to the electronic supplementary material.

Supplementary material 1 (PDF 273 kb)

## Figures and Tables

**Fig. 6:**
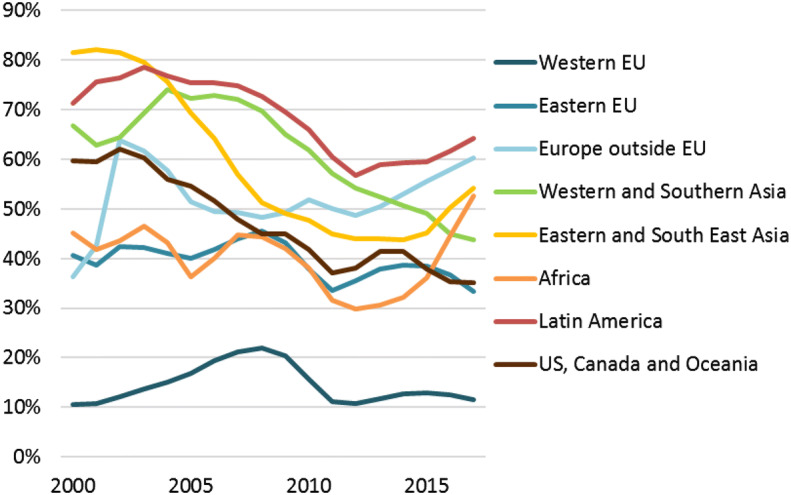
Share of newly arrived immigrant women (age 15–49) in Norway who were registered as family migrants, by origin areas. Per cent. 2000–2017

**Fig. 7:**
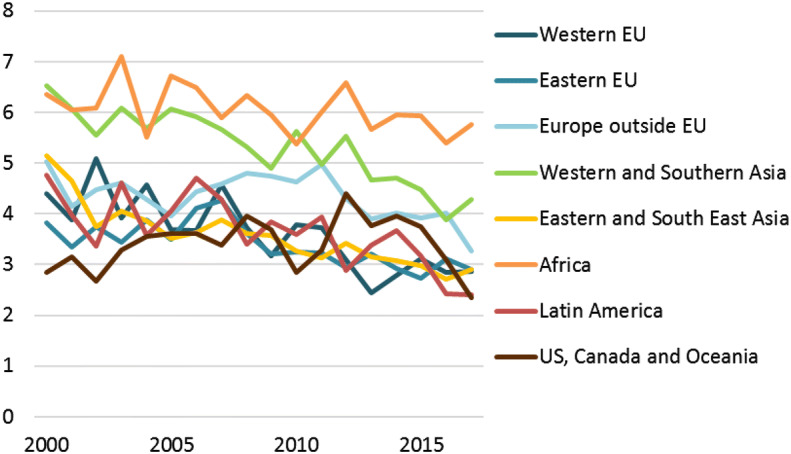
Total fertility rate of newly arrived immigrant women (age 15–49) in Norway who were registered as family migrants, by origin areas. 2000–2017

## Appendix 1: The What-If Scenarios, the Decomposition and the Difference

As shown in Sect. 3.4, the total fertility rate (TFR) in year *t* can be written as

$${\text{TFR}}_{t} = \sum\limits_{a} {\sum\limits_{i} {\left( {{\text{ASFR}}_{ait} \cdot w_{ait} } \right)} }$$

where *a* is age group, *i* is immigrant subgroup, ASFR_*ait*_ are the age-specific fertility rates and *w*_*ait*_ is group *i*’s share of all immigrant women (in that age group). For simplicity, I assume 1-year age groups here. The first what-if scenario is calculated as

$${\text{TFR}}_{t}^{(1)} = \sum\limits_{a} {\sum\limits_{i} {\left( {{\text{ASFR}}_{ai2000} \cdot w_{ait} } \right)} }$$

In other words, the proportion in group *i* is allowed to change while fertility is kept constant. At *t* = 2017, the what-if TFR is $\sum\nolimits_{a} {\sum\nolimits_{i} {{\text{ASFR}}_{ai2000} \cdot w_{ai2017} } }$.The difference between this and the actual fertility in *t* = 2000 is

1 $$\Delta {\text{TFR}}^{(1)} = \sum\limits_{a} {\sum\limits_{i} {{\text{ASFR}}_{ai2000} \cdot \Delta w_{ai} } }$$

where Δ*w*_*ai*_ = *w*_*ai*2017_–*w*_*ai*2000_. Similarly, the second what-if scenario is calculated as

$${\text{TFR}}_{t}^{(2)} = \sum\limits_{a} {\sum\limits_{i} {{\text{ASFR}}_{ait} \cdot w_{ai2000} } }$$

At time *t* = 2017, the difference between this and the actual fertility in *t* = 2000 is

2 $$\Delta {\text{TFR}}^{(2)} = \sum\limits_{a} {\sum\limits_{i} {\Delta {\text{ASFR}}_{ait} \cdot w_{ai2000} } }$$

The real TFR difference, ΔTFR = TFR_2017_–TFR_2000_, is not equal to ΔTFR^(1)^ + ΔTFR^(2)^. Instead, it can be written as

3 $$\Delta {\text{TFR}} = \sum\limits_{a} {\sum\limits_{i} {\left[ {\left( {\overline{{{\text{ASFR}}_{ai} }} \cdot \Delta w_{ai} } \right) + \left( {\Delta {\text{ASFR}}_{ai} \cdot \overline{{w_{ai} }} } \right)} \right]} }$$

where $\overline{{{\text{ASFR}}_{ai} }}$ and $\overline{{w_{ai} }}$ are the mean values $\frac{{{\text{ASFR}}_{ai2000} + {\text{ASFR}}_{ai2017} }}{2}$ and $\frac{{w_{ai2000} + w_{ai2017} }}{2}$, respectively. Equation (3) can be described as a Kitagawa decomposition. Note that

$$\overline{{{\text{ASFR}}_{ai} }} \cdot \Delta w_{ai} = \left( {{\text{ASFR}}_{ai2000} \cdot \Delta w_{ai} } \right) + \left( {\frac{{\Delta {\text{ASFR}}_{ai} \cdot \Delta w_{ai} }}{2}} \right)$$

and

$$\Delta {\text{ASFR}}_{ai} \cdot \overline{{w_{ai} }} = \left( {\Delta {\text{ASFR}}_{ai} \cdot w_{ai2000} } \right) + \left( {\frac{{\Delta {\text{ASFR}}_{ai} \cdot \Delta w_{ai} }}{2}} \right)$$

Therefore, the contribution attributed to changed composition, $\sum\nolimits_{a} {\sum\nolimits_{i} {\left( {\overline{{{\text{ASFR}}_{ai} }} \cdot \Delta w_{ai} } \right)} } ,$ is the same as the difference between the first what-if scenario and actual fertility (Eq. 1) plus $\frac{{\Delta {\text{ASFR}}_{ai} \cdot \Delta w_{ai} }}{2}$ (which is a quite small term). Similarly, the contribution attributed to change in fertility is not given by Eq. (2); the (small) term $\frac{{\Delta {\text{ASFR}}_{ai} \cdot \Delta w_{ai} }}{2}$ is added.

## Appendix 2

See Figs. 6, 7 and Table 2.

## Footnotes

1 Examples include Haug et al. (2002) and Sobotka (2008) for European overviews; Abbasi-Shavazi and McDonald (2000) and Carmichael et al. (2003) for Australia; Zeman et al. (2015) for Austria; Sobotka (2011) for Austria, Germany and Switzerland; Bélanger et al. (2002), Woldemicael and Beaujot (2012) and Adserà and Ferrer (2010, 2011, 2014, 2016) for Canada; Puur et al. (2017) for Russians in Estonia, Toulemon (2004) and Héran et al. (2007) for France; Adserà et al. (2012) for Canada, UK and France; Mayer and Riphahn (2000), Milewski (2007, 2010), Schmid and Kohls (2010), Stichnoth et al. (2013), Cygan-Rehm (2014) and Wolf (2016) for Germany; Mussino and Strozza (2012), Mussino et al. (2015), Ortensi (2015) and Giannantoni and Strozza (2015) for Italy; Goldstein et al. (2009) for Greece, Italy and Spain; Garssen and Nicolaas (2008) and Fokkema et al. (2008) for the Netherlands; Castro Martín and Rosero-Bixby (2011), del Rey and Grande (2015), Kraus and Castro-Martín (2017) and González-Ferrer et al. (2017) for Spain; Andersson (2004), Persson et al. (2010), Persson (2013) and Persson and Hoem (2014) for Sweden; Tromans et al. (2009), Coleman and Dubuc (2010), Dubuc (2012), Waller et al. (2012), Dormon (2014), Wilson (2015), Kulu and Hannemann (2016), Robards and Berrington (2016) and Wilson and Kuha (2017) for UK; Blau and Francine (1992), Kahn (1994), Carter (2000), Lindstrom and Saucedo (2002), Frank and Heuveline (2005), Blau et al. (2008), Parrado (2011), Lichter et al. (2012), Livingston et al. (2012), Choi (2014) and National Academies of Sciences (2015) for the USA; and Mora et al. (2017) for Latinas in USA and Spain.

2 The origin area *Western EU* includes all countries in Western Europe, and Greece and Cyprus (many of the immigrants in this group are from the Nordic countries or Germany), *Eastern EU* includes the 11 new Central and Eastern European EU members since 2004 (Polish and Lithuanian immigrants are large groups here), *Europe outside the EU* includes all non-EU countries in Eastern Europe (many in this group are from Russia or former Yugoslavia), *Western and Southern Asia* includes all Asian countries west of Myanmar/Burma—including Turkey (the Iraqi, Pakistani, Indian, Turkish and Iranians are large groups), *Eastern and South East Asia* includes all Asian countries east of India—including China and Mongolia (large groups here are from Vietnam, Thailand and Philippines), *Africa* includes the whole continent (Somalis and Eritreans are large groups), *Latin America* includes all South and Central America, as well as the Caribbean and Mexico (many are from Chile and Brazil, however, this group is small in Norway), and *USA, Canada and Oceania* includes USA, Canada, New Zealand, Australia and the Pacific islands (also a small group, most are from the USA).

3 Duration of stay is defined as the number of years since (first) migration to Norway.

4 Many subgroups (and the natives) have experienced declined fertility since 2009, which partly may be due to economic uncertainty after the financial crisis (Lappegård et al. 2015; Hart et al. 2015). The what-if scenarios in Fig. 4, lower right panel, suggest that newly arrived immigrants from Western countries contributed to the fertility decline mainly after 2009, which might be a result of economic distress. However, the decline among Non-Western newly arrived immigrants seems relatively unaffected by the financial crisis, as illustrated in Fig. 3.
