# Supplementary material for: Declined Total Fertility Rate Among Immigrants and the Role of Newly Arrived Women in Norway
Source: Eur J Popul. 2019 Nov 6;36(3):547–73. doi: 10.1007/s10680-019-09541-0 (PMC7363761; doi:10.1007/s10680-019-09541-0)
Supplement: Supplementary file 1 — Supplementary material 1 (PDF 273 kb) [file 10680_2019_9541_MOESM1_ESM.pdf]

```
/* Program making what-if-scenarios and the decomposition */

LIBNAME sftmappe '$INNVBRN/wk48/ny2019/sft';
LIBNAME alder  '$INNVBRN/wk48/ny2019/alder';
LIBNAME wk '$INNVBRN/wk48/ny2019';
LIBNAME innvbrn  '$INNVBRN/wk48/ny2019';
LIBNAME mfm '$INNVBRN/wk48/ny2019/mfm';

%LET startar = 2000;
%LET sluttar = 2017;

/* making pooled data sets */
DATA asfrsamle;
SET alder.asfrgruppe111 alder.asfrgruppe112 alder.asfrgruppe113
alder.asfrgruppe114 alder.asfrgruppe121 alder.asfrgruppe122
alder.asfrgruppe123 alder.asfrgruppe124 alder.asfrgruppe131
alder.asfrgruppe132 alder.asfrgruppe133 alder.asfrgruppe134
alder.asfrgruppe141 alder.asfrgruppe142 alder.asfrgruppe143
alder.asfrgruppe144 alder.asfrgruppe151 alder.asfrgruppe152
alder.asfrgruppe153 alder.asfrgruppe154 alder.asfrgruppe161
alder.asfrgruppe162 alder.asfrgruppe163 alder.asfrgruppe164
alder.asfrgruppe171 alder.asfrgruppe172 alder.asfrgruppe173
alder.asfrgruppe174 alder.asfrgruppe181 alder.asfrgruppe182
alder.asfrgruppe183 alder.asfrgruppe184;
RUN;

DATA asfr1; SET asfrsamle; WHERE aldgr='1'; RUN;
DATA asfr2; SET asfrsamle; WHERE aldgr='2'; RUN;
DATA asfr3; SET asfrsamle; WHERE aldgr='3'; RUN;
DATA asfr4; SET asfrsamle; WHERE aldgr='4'; RUN;
DATA asfr5; SET asfrsamle; WHERE aldgr='5'; RUN;
DATA asfr6; SET asfrsamle; WHERE aldgr='6'; RUN;
DATA asfr7; SET asfrsamle; WHERE aldgr='7'; RUN;

DATA kvinnersamle;
SET mfm.mfmgruppe111 mfm.mfmgruppe112 mfm.mfmgruppe113 mfm.mfmgruppe114
mfm.mfmgruppe121 mfm.mfmgruppe122 mfm.mfmgruppe123 mfm.mfmgruppe124
mfm.mfmgruppe131 mfm.mfmgruppe132 mfm.mfmgruppe133 mfm.mfmgruppe134
mfm.mfmgruppe141 mfm.mfmgruppe142 mfm.mfmgruppe143 mfm.mfmgruppe144
mfm.mfmgruppe151 mfm.mfmgruppe152 mfm.mfmgruppe153 mfm.mfmgruppe154
mfm.mfmgruppe161 mfm.mfmgruppe162 mfm.mfmgruppe163 mfm.mfmgruppe164
mfm.mfmgruppe171 mfm.mfmgruppe172 mfm.mfmgruppe173 mfm.mfmgruppe174
mfm.mfmgruppe181 mfm.mfmgruppe182 mfm.mfmgruppe183 mfm.mfmgruppe184;
RUN;

DATA kvinner1; SET kvinnersamle; WHERE aldgr='1'; RUN;
DATA kvinner2; SET kvinnersamle; WHERE aldgr='2'; RUN;
DATA kvinner3; SET kvinnersamle; WHERE aldgr='3'; RUN;
DATA kvinner4; SET kvinnersamle; WHERE aldgr='4'; RUN;
DATA kvinner5; SET kvinnersamle; WHERE aldgr='5'; RUN;
DATA kvinner6; SET kvinnersamle; WHERE aldgr='6'; RUN;
DATA kvinner7; SET kvinnersamle; WHERE aldgr='7'; RUN
```

```
/* WHAT-IF SCENARIOS, FIRST ONLY CHANGED FERTILITY, CONSTANT COMP. */
/* NB: If composition is changed, do also change where marked below */


%MACRO whatif;
%DO ar=&startar. %TO &sluttar.;

      %DO alder = 1 %TO 7;
      DATA nyebarn&alder.;
      MERGE wk.gruppenr asfr&alder. kvinner&alder.;

            /* To be used to change fertility only for sub groups */
            nyebarn&ar.=rate2000*mfm2000/5;
            IF gruppe=111 or gruppe=121 or gruppe=131 or gruppe=181
      /*    IF gruppe=141 or gruppe=151 or gruppe=161 or gruppe=171 */
            THEN
            nyebarn&ar.=rate&ar.*mfm2000/5;

            /* To be used for changing composition and constant fert */
            /* NB! Remember to also change two places below */
      *     nyebarn&ar.=rate2000*mfm&ar./5;

            /* To let everything change like it actually did
            nyebarn&ar.=rate&ar.*mfm&ar./5;*/

      /* Calculate #births and #women, and then the new TFR*/
      PROC MEANS DATA=nyebarn&alder. noprint;
      VAR nyebarn&ar. MFM2000; /*NB: Change to MFM&ar. if composition
is allowed to change!*/
      OUTPUT OUT = sumkvbarn&alder. (drop= _type_ _freq_)
sum(nyebarn&ar.)=sumbarn sum(MFM2000)=sumkv; /* Also here! */

      DATA nyasfr&ar.&alder. (KEEP=nyasfr&alder. sumbarn&alder.);
      SET sumkvbarn&alder.;
      nyasfr&alder.=(sumbarn/sumkv)*5;
      sumbarn&alder.=sumbarn;
      RUN;
      %END;

DATA nysft&ar. (KEEP=nysft&ar.);
MERGE nyasfr&ar.1-nyasfr&ar.7;
nysft&ar. = SUM (OF nyasfr1-nyasfr7);
RUN;

DATA fodte&ar. (KEEP= nybarn&ar.);
MERGE nyasfr&ar.1-nyasfr&ar.7;
nybarn&ar. = SUM (OF sumbarn1-sumbarn7);
RUN;

%END;
```

```
/* Collect the new TFR in a time series*/
DATA nysfttidserie;
MERGE nysft&startar. - nysft&sluttar.;
RUN;

DATA nyebarntidsserie;
MERGE fodte&startar.-fodte&sluttar.;
run;

%MEND whatif;
%whatif;
RUN;
```

```
/* THE DECOMPOSITION */


/* Make common file with all 32x7 groups, dekomp in own columns*/
DATA dekompfil (KEEP= lgr bgr aldgr rate&startar. rate&sluttar.
mfm&startar. mfm&sluttar.);
MERGE wk.lgrbgraldgr asfrsamle kvinnersamle;
IF rate&startar. = '.' THEN rate&startar.=0;
IF rate&sluttar. = '.' THEN rate&sluttar.=0;
RUN;
     /*Calculate total #women*/
     PROC SORT data=dekompfil;
     BY aldgr;
     RUN;

     PROC MEANS DATA=dekompfil noprint;
     VAR mfm&startar. mfm&sluttar.;
     BY aldgr;
     OUTPUT OUT = andelmfm (drop= _type_ _freq_)
     sum(mfm&startar.)=sum&startar. sum(mfm&sluttar.)=sum&sluttar.;
     RUN;

     /*Calculate the shares in each age group*/
     DATA dekompfil;
     MERGE dekompfil andelmfm;
     BY aldgr;
     andel&startar.=mfm&startar./sum&startar.;
     andel&sluttar.=mfm&sluttar./sum&sluttar.;
     RUN;
```

```
/* calculating the effect of overall changed composition (sms) and
changed fertility (frb) */

DATA dekompfil2;
SET dekompfil;
smseffekt=((rate&startar.+rate&sluttar.)/2)*(andel&startar.-
andel&sluttar.);
frbeffekt=((andel&startar.+andel&sluttar.)/2)*(rate&startar.-
rate&sluttar.);
RUN;

PROC MEANS DATA=dekompfil2 noprint;
VAR smseffekt; OUTPUT OUT = smseffekt (drop= _type_ _freq_)
sum(smseffekt)=sumsms; RUN;

PROC MEANS DATA=dekompfil2 noprint;
VAR frbeffekt; OUTPUT OUT = frbeffekt (drop= _type_ _freq_)
sum(frbeffekt)=sumfrb; RUN;


/* effect of changed fertility for each group of duration of stay */
PROC MEANS DATA=dekompfil2 noprint;
WHERE bgr='1';
VAR frbeffekt; OUTPUT OUT = frbeffektbgr1 (drop= _type_ _freq_)
sum(frbeffekt)=bgr1; RUN;

PROC MEANS DATA=dekompfil2 noprint;
WHERE bgr='2';
VAR frbeffekt; OUTPUT OUT = frbeffektbgr2 (drop= _type_ _freq_)
sum(frbeffekt)=bgr2; RUN;

PROC MEANS DATA=dekompfil2 noprint;
WHERE bgr='3';
VAR frbeffekt; OUTPUT OUT = frbeffektbgr3 (drop= _type_ _freq_)
sum(frbeffekt)=bgr3; RUN;

PROC MEANS DATA=dekompfil2 noprint;
WHERE bgr='4';
VAR frbeffekt; OUTPUT OUT = frbeffektbgr4 (drop= _type_ _freq_)
sum(frbeffekt)=bgr4; RUN;


/* among the newly arrived: effect of changed fertility for each origin
country group */

PROC MEANS DATA=dekompfil2 noprint;
WHERE bgr='1' and lgr='1';
VAR frbeffekt; OUTPUT OUT = frbeffektlgr1 (drop= _type_ _freq_)
sum(frbeffekt)=lgr1; RUN;

PROC MEANS DATA=dekompfil2 noprint;
WHERE bgr='1' and lgr='2';
```

```
VAR frbeffekt; OUTPUT OUT = frbeffektlgr2 (drop= _type_ _freq_)
sum(frbeffekt)=lgr2; RUN;

PROC MEANS DATA=dekompfil2 noprint;
WHERE bgr='1' and lgr='3';
VAR frbeffekt; OUTPUT OUT = frbeffektlgr3 (drop= _type_ _freq_)
sum(frbeffekt)=lgr3; RUN;

PROC MEANS DATA=dekompfil2 noprint;
WHERE bgr='1' and lgr='4';
VAR frbeffekt; OUTPUT OUT = frbeffektlgr4 (drop= _type_ _freq_)
sum(frbeffekt)=lgr4; RUN;

PROC MEANS DATA=dekompfil2 noprint;
WHERE bgr='1' and lgr='5';
VAR frbeffekt; OUTPUT OUT = frbeffektlgr5 (drop= _type_ _freq_)
sum(frbeffekt)=lgr5; RUN;

PROC MEANS DATA=dekompfil2 noprint;
WHERE bgr='1' and lgr='6';
VAR frbeffekt; OUTPUT OUT = frbeffektlgr6 (drop= _type_ _freq_)
sum(frbeffekt)=lgr6; RUN;

PROC MEANS DATA=dekompfil2 noprint;
WHERE bgr='1' and lgr='7';
VAR frbeffekt; OUTPUT OUT = frbeffektlgr7 (drop= _type_ _freq_)
sum(frbeffekt)=lgr7; RUN;

PROC MEANS DATA=dekompfil2 noprint;
WHERE bgr='1' and lgr='8';
VAR frbeffekt; OUTPUT OUT = frbeffektlgr8 (drop= _type_ _freq_)
sum(frbeffekt)=lgr8; RUN;
```
